# A novel LC-MS/MS method for the determination of favipiravir ribofuranosyl-5’-triphosphate (T-705-RTP) in human peripheral mononuclear cells

**DOI:** 10.1016/j.jpba.2024.116155

**Published:** 2024-04-21

**Authors:** Elizabeth Challenger, Sujan Dilly-Penchala, Colin Hale, Richard Fitzgerald, Helen Reynolds, Justin Chiong, Tim Rowland, Tom Fletcher, Saye Khoo, Laura Else

**Affiliations:** a Department of Pharmacology and Therapeutics, Institute of Systems and Molecular Biology, University of Liverpool, Liverpool L7 8TX, UK; b Liverpool University Hospitals NHS Foundation Trust, Prescot Street, Liverpool L7 8XP, UK; c Liverpool School of Tropical Medicine, Pembroke Place, Liverpool L3 5QA, UK

**Keywords:** T-705 RTP, Favipiravir RTP, SARS-CoV-2, COVID-19, Bioanalysis

## Abstract

Favipiravir is a broad-spectrum antiviral that is metabolised intracellularly into the active form, favipiravir ribofuranosyl-5’-triphosphate (F-RTP). Measurement of the intracellular concentration of F-RTP in mononuclear cells is a crucial step to characterising the pharmacokinetics of F-RTP and to enable more appropriate dose selection for the treatment of COVID-19 and emerging infectious diseases. The described method was validated over the range 24 – 2280 pmol/sample. Peripheral blood mononuclear cells (PBMCs) were isolated from whole blood and lysed using methanol-water (70:30, v/v) before cellular components were precipitated with acetonitrile and the supernatant further cleaned by weak anion exchange solid phase extraction. The method was found to be both precise and accurate and was successfully utilised to analyse F-RTP concentrations in patient samples collected as part of the AGILE CST-6 clinical trial.

## 1 Introduction

Favipiravir (FVP) is a pyrazine carboxamide derivative that is licenced in Japan to treat influenza and has been shown to have activity against several viral families, including arenaviruses, flaviviruses, filoviruses and coronaviruses [1]. FVP is a prodrug that is ribosylated and phosphorylated intracellularly to the active favipiravir ribofur-anosyl-5’-triphosphate (F-RTP), which is a substrate for viral RNA dependent RNA polymerase (RdRp) and is incorporated into the nascent RNA chain, inducing chain termination or lethal mutagenesis. Due to its broad-spectrum activity, oral FVP has been investigated and utilised as a repurposed therapeutic for treatment of Ebola virus during the 2014–2015 outbreak in West Africa and most recently, to treat severe acute respiratory syndrome coronavirus-2 (SARS-CoV-2) infection in Asia [2,3].

FVP has demonstrated activity against SARS-CoV-2 *in vitro* [4], however clinical trials investigating FVP have so far been inconclusive. While early clinical studies indicated oral FVP to be efficacious in individuals with mild to moderate COVID-19 disease [5–7], more recent data from randomised controlled trials suggests that oral FVP does not substantially improve clinical outcomes in patients with COVID-19. In the PIONEER trial, 251 patients were randomised to receive FVP plus standard of care (SoC) with a further 248 patients receiving SoC only. Patients in the FVP arm received 1800 mg twice daily on day 1 on treatment, followed by 800 mg twice daily for a further 9 days. The trial concluded that there was no significant difference in time to recovery between patients who received FVP and those who received SoC alone. Post-hoc analysis indicated there may be faster recovery in patients below 60 years of age, but use of FVP should be with caution until further data becomes available [8].

The lack of virologic efficacy and symptom improvement as seen in these studies has been attributed to the dose of FVP administered, which is the same used to treat influenza virus, as well as lower than expected plasma FVP concentrations. Indeed, *in vitro* evidence implies that higher doses may be required to effectively treat SARS-CoV-2. The use of a novel intravenous formulation of FVP is postulated to overcome the previous issues encountered; bypassing first pass metabolism may achieve higher plasma concentrations of FVP, and therefore of the intracellular active metabolite (F-RTP), resulting in more pronounced efficacy.

The complex, non-linear pharmacokinetics (PK) of favipiravir and wide ranging 50 % effective concentration (EC_50_) adds difficulty to identifying the appropriate dosing regimen for emerging infectious diseases such as COVID-19. For SARS-CoV-2, the reported EC_50_ of favipiravir ranges from 62 to over 500 µM (10 to > 78 μg/mL) [9]. Given that FVP is approximately 54 % protein bound, studies investigating FVP for the treatment of COVID-19 have targeted a C_min_ of 20 μg/mL [10]. Variability in FVP PK has been observed across animal species, with day 7 average plasma concentrations 19 – 54 % lower in Mauritian cynomolgus macaques compared to Chinese cynomolgus macaques [11] and in human trials investigating FVP for EBOV infection, where plasma concentrations are reported to be 50 % lower in patients from the United States than patients from Japan [11]. Similarly, the JIKI trial investigated FVP for the treatment of EBOV disease and found that, although FVP was well tolerated, results were not definitive regarding safety or improvement of clinical outcomes [12]. This may be due to plasma concentrations not achieving target exposure, variability between patients and unexpected reduction in plasma concentrations that were of undefined causes [2].

In order to assist with appropriate dose selection for COVID-19, it would be beneficial to ascertain *in vivo* concentrations of F-RTP at the site of action, within respiratory tissues. Human peripheral blood mononuclear cells (PBMC) are considered as a viable alternative to tissue biopsy, given the availability of peripheral blood and ethical and practical difficulties of conducting tissue biopsy of the respiratory tract [13]. As F-RTP is formed in PBMC, quantitative measurements in PBMC may serve as a surrogate for F-RTP tissue exposures at the site of action (e.g. lung tissue). At the time of writing, there are very limited PK data for F-RTP. The reported terminal half-life (t_1/2_) of F-RTP in PBMC is approximately 2 h, compared to 4.2 h in the lung, and the therapeutic effect of FVP is thought to be dependent on maintaining a minimum concentration of F-RTP in the tissue [14].

The AGILE CST-6 trial (EudraCT 2020–001860–27) investigated multiple ascending doses of FVP given intravenously (IV) to hospitalised patients with polymerase chain reaction (PCR) confirmed COVID-19. IV FVP was infused for 1 h every 12 h for 7 days, and PBMC samples were collected between 6 and 12 h following completion of the first infusion on days 1, 3 and 5.

Here we describe the preliminary validation of an LC-MS method for quantification of F-RTP in PBMC in patients receiving IV FPV as part of the AGILE CST-6 clinical trial.

## 2 Materials and methods

### 2.1 Chemicals

F-RTP Sodium Salt and the internal standard, tenofovir-d6-diphosphate (TFV-d6-DP), were purchased from Toronto Research Chemicals, Ontario, Canada. An Avidity Duo system (Avidity Science, Buckinghamshire, UK) supplied ultra-pure (18 Ω) water. Ammonium acetate, formic acid and ammonium hydroxide were purchased from Merck (Gillingham, UK). Methanol and acetonitrile of LC-MS grade were acquired from Fisher Scientific (Loughborough, UK). PBMC were isolated from drug-free whole blood obtained from the National Health Service Blood and Transplant service (Liverpool, UK). Ethical approval was granted by the NHS Health Research Authority.

### 2.2 Equipment

The system comprised a Shimadzu Nexera® X2 uHPLC (pumps, column oven, autosampler; Milton Keynes, UK) coupled to an AB Sciex 5500 triple quadrupole mass spectrometer with a heated electrospray ionisation (H-ESI) source (AB Sciex, Framingham, MA, USA). Column oven and autosampler temperatures were set at 40°C and 4°C, respectively. A ThermoFisher Biobasic AX™ 50 × 1 mm, 5 µm column was used for separation and elution. Waters (Wilmslow, UK) OASIS weak anion exchange (WAX) solid phase extraction (SPE) cartridges (1cc, 30 mg/mL) were used for sample preparation. Data was acquired using Analyst (v1.6.1) and quantified using MultiQuant (v3.0.3) (AB Sciex).

### 2.3 PBMC isolation for calibrators and quality control samples

For the preparation of calibrators and quality control (QC) samples, PBMC were isolated from drug-free whole blood collected from healthy volunteers via the NHS Blood and Transplant Service. A 50 mL Falcon tube was filled with 10 mL Ficoll Paque Plus (Cytiva, Buckinghamshire, UK). Whole blood (20 mL) was carefully added to the tube, such that the whole blood rested on top of the Ficoll. Tubes were centrifuged at 671 *x g* for 30 min, with no deceleration brake. The plasma layer was carefully removed using Pasteur pipettes and discarded. The layer at the interface (i.e. buffy coat) was transferred into clean a 50 mL Falcon tube using a Pasteur pipette. Ice cold Hanks’ balanced salt solution (HBSS; Merck) was added to a final volume of 50 mL and tubes centrifuged at 671 *x g* for 5 min. Supernatant was discarded before reconstitution of the pellet in 10 mL of HBSS and cell counting. Tubes were re-centrifuged at 671 *x g* for 5 min and supernatant was discarded. In order to lyse the cells, the pellet was resuspended in 477.5 mL of methanol-water (70:30, v/v) to obtain a final cell count of 2 × 10^6^ cells/mL (total cell count 954,750,000).

### 2.4 Stock solution preparation

F-RTP Sodium Salt stock powder was diluted in methanol-0.5 % formic acid in water (95:5, v/v) to a final concentration of 950 µM. Intermediate dilutions were prepared by dilution of the stock solution in methanol-water (70:30, v/v). Nine intermediate solutions for the calibration curve were generated at the following concentrations: 114000, 57000, 40014, 30011, 19987, 11992, 3981, 2389 and 1194 nM. QC intermediate solutions were made at concentrations of 60002, 32305, 2519 and 1194 nM. Intermediate solutions were spiked into cell lysate (20 μL into 1 mL containing 2 × 10^6^ cells) to produce a calibration curve with the following concentration levels: 2280, 1140, 800, 600, 400, 240, 70, 48, and 24 pmol/sample (1 mL containing 2 × 10^6^ cells). Quality control samples were spiked in the same manner to a final concentration of 1300, 700, 55 and 24 pmol/sample for high, medium, low and lower limit of quantification QCs, respectively.

Internal standard (IS) stock solution was made to a concentration of 2 mM by diluting 1 mg of TFV-d6-DP in 900 µL of water. Working IS solution was made by diluting the stock 1 in 100 with methanol-water (70:30, v/v) to a final concentration of 20 µM.

### 2.5 Extraction procedure

The volume of PBMC lysate used was calculated so that the on-column sample contained 2 × 10^6^ cells/mL. For the calibrators and QCs, this equated to 1 mL. To all samples, 1 mL of acetonitrile containing 2 % formic acid was added, followed by 20 µL of IS (TFV-d6-DP, 20 µM). Samples were vortex mixed and centrifuged at 2688 *x g*. SPE cartridges were conditioned with 1 mL of 100 % methanol and centrifuged for 1 min at 377 *x g*. The SPE cartridges were further conditioned with water-methanol-formic acid (73:25:2, v/v/v) and centrifuged. Cartridges were transferred into clean borosilicate tubes and loaded with 1 mL of sample and centrifuged. This step was repeated for any remaining sample volume. SPE cartridges were washed with 1 mL of deionised water, followed by methanol-water (50:50, v/v). Finally, the cartridges were removed into clean borosilicate tubes before elution with 1 mL of acetonitrile-water-ammonium hydroxide (73:25:2, v/v/v). Eluate was dried under nitrogen flow overnight at ambient temperature and reconstituted in 200 µL of methanol-water (70:30, v/v).

### 2.6 LC-MS conditions

A ThermoFisher Biobasic AX™ column (5 µm; 50 × 1 mm) was used for separation with 10 mM ammonium acetate-acetonitrile (70:30, v/v) adjusted to pH 5.5 with acetic acid as mobile phase A and 20 mM ammonium acetate-acetonitrile (70:30, v/v) adjusted to pH 10.5 with ammonium hydroxide solution as mobile phase B. A gradient method with a flow rate of 0.25 mL/min was used. Initial conditions of 15 % B were held for 0.5 min, increased to 90 % B for a further 3 min, and then held at 100 % B for 3 min before returning to 10 % B for 5.5 min to reequilibrate the column at more acidic pH, for a total run time of 12 min. The column conditioning step with low %B is essential for retention of the analyte on the Biobasic™ column. The optimised mass spectrometer settings are summarised in Table 1.

## 3 Validation methodology

This preliminary set of validation experiments was conducted considering guidance from European Medicines Agency (EMA; 2012) and US Food and Drug Administration (FDA; 2018) guidelines [15,16]. Matrix and recovery experiments were performed as detailed by Matuszewski *et al*. [17].

Although the ICH M10 guidelines are now in use for bioanalytical method validation, the experiments detailed were conducted during a phase between the ICH M10 being published and adoption of the guidelines by our laboratory. The validation commenced using the EMA and FDA procedures and was therefore completed using those guidelines.

### 3.1 Precision and accuracy

The precision and accuracy of the assay was assessed by analysis of calibration curve with LLOQ, LQC, MQC and HQC samples in quadruplicate run over a period of five days.

### 3.2 Matrix and recovery effects

Matrix and recovery were both established using methods described by Matuszewski et al. [17]. A single lot of whole blood was used due to the high volume of whole blood needed to generate significant enough numbers of isolated PBMC for lysis to obtain a final cell count of 2 × 10^6^ cells/mL. Extracted LQC, MQC and HQC samples were prepared (n =6) alongside a further six of each QC level prepared by spiking of F-RTP into final reconstitution solution (methanol-water, 70:30, v/v) and into extracted blank PBMC. For recovery, the peak area response of the non-extracted (aqueous) samples was compared to that of samples pre-spiked with F-RTP prior to extraction. The matrix effect was assessed by comparing the peak area response of non-extracted QCs in reconstitution solution to that of F-RTP spiked into blank PBMC extract at each QC level (post-extraction spiked samples).

### 3.3 Stability

The stability of F-RTP in whole blood prior to PBMC isolation was established using incurred samples. Patients enrolled onto the CST-6 trial (refer to Clinical Application) provided consent for a second cell preparation tube (CPT) sample to be collected concurrently with the trial CPT sample. The first tube (t = 0 h; control) was inverted to allow mixing of the blood with anticoagulant and centrifuged as soon as practicable following blood draw (average time to centrifugation = 15 min) and PBMCs isolated, counted, lysed and stored at −80°C. The second CPT (t = 1 h; stability) was inverted to mix whole blood and anticoagulant and incubated at room temperature for 1 h before centrifugation and PBMC isolation, counting, lysis and storage at −80°C. Control and stability samples were analysed together with a calibration curve and the percentage difference of the calculated concentration was evaluated. Reinjection reproducibility was determined by re-injection of an accepted precision and accuracy experiment after 24 h storage in the autosampler at 4°C. Testing is ongoing to establish the long-term stability of F-RTP in spiked PBMC lysate.

### 3.4 Clinical application

The described method was developed for the AGILE CST-6 clinical trial – a randomised, multicentre, seamless, adaptive, phase I/II platform study to determine the phase II dose and evaluate safety and efficacy of intravenous (IV) FVP for the treatment of COVID-19 (EudraCT 2020–001860–27). Patients admitted to hospital with PCR-confirmed SARS-CoV-2 infection with severe COVID-19 requiring oxygen therapy by mask or non-invasive high-flow ventilation were enrolled within 14 days of the onset of symptoms and randomised (2:1) to receive FVP or the standard of care (n=6 per cohort; 4 FVP: 2 SoC). FVP was administered by IV infusion over 1 h, given every 12 h for seven days. The study involved increasing doses of FVP, beginning with 600 mg BID with dose escalation directed by the study Safety Review Committee. Whole blood was collected in CPT for the isolation of PBMC on days one, three and five between 6- and 12-h following the completion of the infusion. CPT were centrifuged immediately following collection and the PBMC layer isolated, cells counted and subsequently lysed in 1 mL of methanol-water (70:30, v/v) before storage at −80°C until analysis. At the time of LC-MS analysis, a given volume of PBMC lysate was taken to achieve an on-column count of 2 × 10^6^ cells and extracted as described above. Where cell counts were below 2 × 10^6^ cells/mL, the entire sample was used (1 mL).

The pharmacokinetic data are to be presented in a separate manuscript. A subset of the initial data (600 mg cohort – day 3 only; n= 4 patients) are presented descriptively to support the clinical application of the analytical method.

## 4 Results

### 4.1 LC-MS conditions

Negative ion mode using multiple reaction monitoring (MRM) was used for the detection of F-RTP. Two *m/z* transitions were initially monitored for F-RTP – 527.9 → 272.8 and 527.862→ 430.0 however, we observed non-linearity of the calibration curve when using the product ion *m/*z 430.0 and therefore the validated method utilised 527.9 → 272.8. TFV-d6-DP transition monitored was 451.948 → 354.0.

**Fig. 1:**
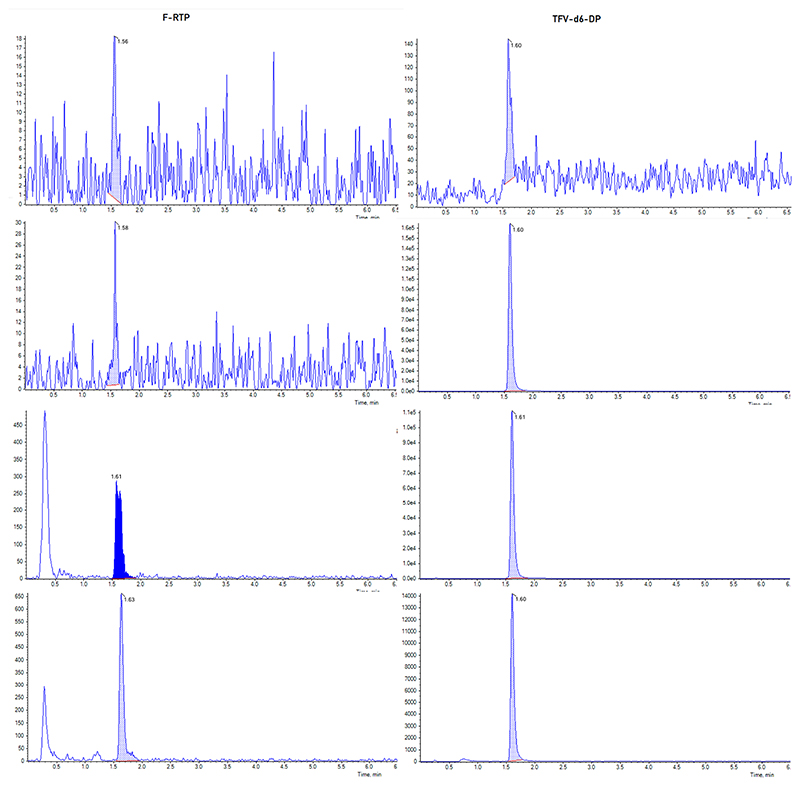
F-RTP chromatograms from extracted PBMC samples A) double blank sample (no IS); B) blank with IS; C) LLOQ and IS; D) patient sample (186.4 pmol/106 cells) and IS.

F-RTP eluted from the column at 1.57 min and TFV-d6-DP eluted at 1.60 min, at 90 % mobile phase B. Example chromatograms from blank, internal standard, lower limit of quantification, high quality control and a clinical sample are shown in Fig. 1.

A pre-equilibration step that involved conditioning the column conditioning for 60 min with mobile phase A was needed in order to retain the analytes on column. It was also necessary to include an extended re-equilibration step at the end of the gradient program to reacidify the stationary phase of the column for the subsequent injection.

### 4.2 Precision and accuracy

The method was found to be both precise and accurate, with inter- and intra-assay assessments within ± 20 % for the LLOQ QC and within ± 15 % of the nominal concentrations for LQC, MQC and HQC samples (Table 2). Quadratic 1/x^2^ regression was utilised to produce the best fit for the concentration-detector response, with an average correlation coefficient (r^2^) of 0.99511 (n=3).

### 4.3 Recovery and matrix effect

Matrix effect, recovery and process efficiency were assessed using LQC, MQC and HQC samples (n =6). The results are presented in Table 3. Matrix effect (ME) was 83 % across the three concentrations analysed whilst extraction recovery (RE) and process efficiency (PE) were 38 % and 33 %, respectively. Although RE and PE are low, experimental results are consistent, with coefficient of variation (%CV) values < 15 % for all conditions. The calculated RE and PE are also consistent with similar methods, such as that reported for detection of ribavirin triphosphate in PBMC [18].

### 4.4 Stability

The stability of F-RTP was evaluated using a total of 4 sample pairs collected from three patients enrolled on AGILE CST-6 receiving IV FPV (600 mg) on day 3 or day 5. F-RTP was found to be unstable in liquid whole blood collected in CPT, when left at ambient temperature for approximately 1 h (Table 4). The % decrease in F-RTP concentration was variable and was on average (range) 61 % (17–85 %). Extracted samples reinjected after 24 h in the autosampler were found to be stable with %bias < 12 % and %CV < 4 % for all concentrations tested.

### 4.5 Clinical application

Fig. 1 depicts a chromatogram from an extracted PBMC sample from a patient receiving IV FVP (600 mg BID, Day 3). All of the samples collected for the 600 mg cohort were quantifiable, with F-RTP concentrations on day 3, collected between 6 and 12 h post first IV FVP infusion, ranging between 60.6 – 186.4 pmol/10^6^ cells.

## 5 Discussion

A selective, accurate and precise LC-MS/MS method has been developed for the measurement of F-RTP in human peripheral blood mononuclear cells. To the best of our knowledge, this is the first report of a method for quantifying F-RTP itself in PBMC. However, a recent publication described the quantitation of surrogates of favipiravir metabolites using two-dimensional liquid chromatography [19]. The described method offers a relatively simple sample preparation method using weak anion exchange solid phase extraction. The method is a direct quantification method, which does not involve complex dephosphorylation stages that can be time-consuming and laborious.

Positive electrospray ionisation (ESI) mode is generally preferred for analysis of phosphorylated metabolites as positive ESI offers a greater level of specificity. This is largely due to generation of the diphosphate ion (*m/z* 159) in negative mode, which often appears as the most abundant product ion. Several attempts were made to establish this method in positive ESI mode, however F-RTP signal was of poor intensity during tuning and completely absent during analysis of extracted samples. This phenomenon has been noted for triphosphate compounds, such as stavudine and zidovudine triphosphate, where the base moieties only contain ring nitrogen molecules, rather than amino related nitrogen molecules, which are well ionised in positive ESI mode [20]. Given that FVP has a higher number of ring nitrogen molecules than amino-associated nitrogen molecules, this phenomenon may explain the poor signal seen for F-RTP fragmentation in positive ionisation mode compared to negative ionisation mode. Our method therefore proceeded in the negative ionisation mode utilising product ions other than the characteristic diphosphate transition (*m/z* 159) to avoid any interference from endogenous phosphate compounds.

At the time of validation, the availability of F-RTP stock powder was extremely limited, owing to the difficulty in synthesising the compound. This made it challenging to perform precision and accuracy experiments with a greater number of replicate QC samples than described here. Whilst EMA guidelines recommend that HQC samples are at least 75 % of the highest calibrator, the FDA guidelines do not stipulate such a requirement. The QC samples were continuously monitored throughout the validation and beyond, with no observed precision or accuracy concerns. Similarly, despite efforts to procure F-RTP stable isotopically labelled (SIL) internal standard (F-RTP-^13^C_5_), the synthesis also proved difficult for multiple commercial providers. Our method therefore uses TFV-d6-DP as this was the only phosphorylated internal standard that was readily available at the time of validation. It is anticipated that substitution of TFV-d6-DP with F-RTP-^13^C_5_ could offer improvements in assay robustness and matrix effect, and it is our intention to incorporate an F-RTP SIL as and when this becomes commercially available.

A single lot of whole blood was used to isolate and lyse PBMC for calibrator/QC preparation. Ideally, multiple lots of blood would be assessed for selectivity and matrix effect, however due to the volume of blood required (>200 mL) and ethical approval limitations, this was not possible at the time of conducting these experiments. Freeze/thaw assessments were not performed as calibrators and QC samples were spiked fresh into lysate on the day of analysis. Clinical samples, due to low sample volumes, did not undergo repeat analyses to conduct incurred sample reanalysis (ISR) experiments. In many cases, the cell count of the clinical sample was such that the entire sample was exhausted in order to obtain the best “on-column” cell count and therefore opportunities to perform ISR were extremely limited. Establishing freeze/thaw would be beneficial to further application of this method. More detailed data regarding the stability of F-RTP and precision and accuracy of this method will be sought in the future, as the application of the method increases.

Our stability data suggest that F-RTP is unstable in whole blood left at room temperature in CPT. We observed significant degradation of F-RTP in 1 h of up to 85 %, which reinforces the requirement for immediate processing of PBMC samples at clinical sites. Phosphorylated metabolites are unstable in the extracellular environment due to degradation by phosphatases [21], which limits the use of extracellularly spiked samples to infer F-RTP stability in biological matrices. Assessing the stability of F-RTP using incurred whole blood collected from patients allowed for more accurate representation of the intracellular metabolite, rather than direct spiking of extracellular F-RTP solution into whole blood or PBMC lysate. However, this resulted in only a small number of samples available to assess F-RTP stability.

Further analysis is also required to determine if this is enzymatic degradation from phosphatases present in the whole blood or because of environmental storage of the CPT. It has been noted that refrigeration of CPT may be required after mixing of blood with anticoagulant, to prevent degradation of nucleotides at room temperature. However, this is generally considered for intervals of greater than 30 min between specimen collection and centrifugation [20]. CPT collection to centrifugation times recorded for the AGILE CST-6 trial were 15 min on average, and therefore within the reported 30 min time frame before refrigeration should be considered.

Clinical samples from the AGILE CST-6 cohort were analysed using pmol/sample calibration curve and F-RTP concentrations expressed as pmol/10^6^ cells using the cell counts provided by the trial laboratory. F-RTP was quantifiable in all PBMC samples collected from patients receiving a 600 mg dose of IV FVP between 6 and 12 h after the first infusion. F-RTP concentrations on Day 3 were between 60.6 and 186.4 pmol/10^6^ cells, and “on-column” concentrations were within the mid-range of the calibration curve, thereby supporting the ongoing use of this validated calibration range for ascending doses of FVP in the AGILE CST-6 trial, or for other disease indications. Although measured F-RTP levels in clinical cohorts are lacking, PBPK models have been used to estimate F-RTP concentrations from FVP plasma exposures [22]. In order to make comparisons between the clinical samples and the PBPK predicted F-RTP concentrations, the pmol/10^6^ values were converted to μM, using 0.4 pL as the volume of a single PBMC [23]. F-RTP concentrations expressed as pmol/10^6^ cells were divided by the volume of 1 million cells (i.e. 400,000 pL = 0.4 μL) to convert to pmol/μL, equivalent to μmol/L or μM. This yielded significantly higher F-RTP values than predicted by PBPK modelling, but this is likely due to the model using Madin-Darby Canine Kidney (MDCK) cells, which have a much larger volume than PBMC (2.08 pL vs. 0.4 pL) and therefore a lower number of cells per microlitre.

Further work is ongoing to quantify F-RTP in dried blood spots (DBS). Results of these experiments may shed further light on enzymatic degradation of F-RTP in whole blood, as enzymatic activity is halted in DBS samples compared to liquid whole blood stored on the benchtop at room temperature [24]. Comparison of F-RTP in DBS and PBMC samples may provide additional insights on the stability of F-RTP. It may also be of interest in the future to assess whether FVP itself is detectable in PBMC and DBS.

In conclusion, an LC–MS method has been optimized and validated for quantification of F-RTP in human PBMC. The assay was successfully used to quantify F-RTP in clinical samples obtained from patients enrolled on the AGILE CST-6 clinical trial.

## Figures and Tables

**Table 1:** Summary of mass spectrometer parameters.

Parameter	F-RTP	TFV-d6-DP
Mass transition (Da)	527.9→272.8	451.9→354.0
Collision Energy (V)	-38	-30
Spray Voltage (ISV)	-4500	-4500
Vaporizer Temperature (TEM°C)	500	500
Ion Source Gas 1 (GS1)	50	50
Ion Source Gas 2 (GS2)	50	50
Collision Gas (CAD)	8	8
Curtain Gas (CUR)	30	30

**Table 2:** Summary of precision and accuracy validation experiments.

	LLOQ (24 pmol/sample)					LQC (55 pmol/sample)					MQC (700 pmol/sample)					HQC (1300 pmol/sample)			
	Mean	SD	CV (%)	Bias (%)		Mean	SD	CV (%)	Bias (%)		Mean	SD	CV (%)	Bias (%)		Mean	SD	CV (%)	Bias (%)
Inter- day	25.02	2.38	9.51	-1.44		54.85	5.28	9.63	4.79		682.97	67.86	9.94	3.99		1274.91	83.76	6.57	2.10
Intra- day	24.28	0.62	2.54	1.16		56.95	1.06	1.87	3.54		718.36	12.77	1.78	2.62		1316.64	19.95	1.52	1.28

**Table 3:** Results of matrix and recovery validation experiment. A) peak area ratio of aqueous mobile phase solutions without matrix or extraction; B) peak area ratio of analyte spiked into extracted blank samples; C) peak area ratio of analyte spiked into matrix.

Nominal QC Concentration	Mean peak area ratio			ME (%) B/A	Ext RE (%) C/B	PE (%) C/A
	A	B	C			
55	0.059	0.052	0.022	88.5	42.0	37.1
700	0.722	0.598	0.229	82.8	38.3	31.7
1300	1.486	1.172	0.438	78.9	37.4	29.5

**Table 4:** Summary of stability experiment results. The [F-RTP] was assessed in an immediately processed sample (control) and a sample where the whole blood was left for 1 h in CPT before isolating the PBMC (stability). *Unquantifiable F-RTP; assigned a value of 1/2 to establish instability.

Sample ID	Collection Day	Time post infusion (h)	Sample Type	F-RTP Concentration (pmol/106 cells)	% decrease
1	D3	6.05	Control	157.1	17 %
			Stability	131.0	
1	D5	6.25	Control	127.9	85 %
			Stability	18.9	
6	D3	6.50	Control	65.2	82 %
			Stability	12.0*	
12	D5	6.27	Control	50.0	59 %
			Stability	20.4	
				Min	17 %
				Max	85 %
				Mean	61 %
				St Dev	0.31
				CV %	51.68

## Data Availability

Data will be made available on request.
